# Improving load frequency controller tuning with rat swarm optimization and porpoising feature detection for enhanced power system stability

**DOI:** 10.1038/s41598-024-66007-y

**Published:** 2024-07-02

**Authors:** Pasala Gopi, N. Chinna Alluraiah, Pujari Harish Kumar, Mohit Bajaj, Vojtech Blazek, Lukas Prokop

**Affiliations:** 1 Department of Electrical and Electronics Engineering, Annamacharya University, Rajampet, India; 2 School of Electrical and Communication Sciences, JSPM University, Pune, India; 3 Department of Electrical Engineering, Graphic Era (Deemed to be University), Dehradun, 248002 India; 4 Hourani Center for Applied Scientific Research, Al-Ahliyya Amman University, Amman, Jordan; 5 Graphic Era Hill University, Dehradun, 248002 India; 6 ENET Centre, VSB—Technical University of Ostrava, 708 00 Ostrava, Czech Republic

**Keywords:** Porpoising, PID control schemes, Rat swarm optimization, Load frequency control, Firefly algorithm, Automatic generation control (AGC), Energy science and technology, Engineering, Mathematics and computing

## Abstract

Load frequency control (LFC) plays a critical role in ensuring the reliable and stable operation of power plants and maintaining a quality power supply to consumers. In control engineering, an oscillatory behavior exhibited by a system in response to control actions is referred to as “Porpoising”. This article focused on investigating the causes of the porpoising phenomenon in the context of LFC. This paper introduces a novel methodology for enhancing the performance of load frequency controllers in power systems by employing rat swarm optimization (RSO) for tuning and detecting the porpoising feature to ensure stability. The study focuses on a single-area thermal power generating station (TPGS) subjected to a 1% load demand change, employing MATLAB simulations for analysis. The proposed RSO-based PID controller is compared against traditional methods such as the firefly algorithm (FFA) and Ziegler-Nichols (ZN) technique. Results indicate that the RSO-based PID controller exhibits superior performance, achieving zero frequency error, reduced negative peak overshoot, and faster settling time compared to other methods. Furthermore, the paper investigates the porpoising phenomenon in PID controllers, analyzing the location of poles in the s-plane, damping ratio, and control actions. The RSO-based PID controller demonstrates enhanced stability and resistance to porpoising, making it a promising solution for power system control. Future research will focus on real-time implementation and broader applications across different control systems.

## Introduction

Load frequency control (LFC) controllers play a critical role in the smooth and dependable operation of power systems. These controllers are essential for maintaining the stability of the system by regulating its frequency, which is crucial for ensuring the synchronized operation of various interconnected components. In addition to frequency regulation, LFC controllers are responsible for balancing the generation and load within the system. By continuously adjusting the power output of generators in response to changes in demand or disturbances, LFC controllers help to match supply with demand, thereby ensuring grid stability. Moreover, LFC controllers contribute to the overall stability of power plants by mitigating the impact of sudden load changes or fluctuations in renewable energy sources. Compliance with regulatory standards is another vital function of LFC controllers, as they ensure that the power system operates within specified frequency and voltage limits. Without effective LFC controllers, power systems would be more vulnerable to disruptions, such as blackouts or brownouts, voltage fluctuations, frequency deviations, and other reliability issues, which could have significant economic and societal impacts. Therefore, the development and implementation of robust LFC controllers are essential for ensuring the reliable and secure operation of modern power systems.

### Literature on LFC controllers

The Ziegler-Nichols method^1^ is a popular technique for tuning the Proportional-Integral-Derivative (PID) controllers, including those used in LFC^2^. Åström and Hägglund's^3^ work on PID tuning encompasses both theoretical insights and practical methodologies, contributing to the widespread adoption and effective implementation of PID controllers in various industrial processes, including LFC. In addition to these traditional approaches, internal model control (IMC), Tyreus-Luyben (TL), and Cohen-Coon (CC) methods are also employed for assessing the LFC controller settings. However, these approaches have limitations, such as exhibiting aggressive or oscillatory responses, and they may not adequately consider the nonlinearities or uncertainties of the system^4^. The intelligent and nature-inspired methods provide alternate approaches to LFC controller tuning, especially in situations where conventional methods may be time-consuming or unfeasible. These techniques use AI and mimic natural phenomena, resulting in enhanced control performance and efficiency across different industrial applications^5^. The researchers in Ref.^6^ examined the gains of a PID controller for single-area AGC using the Stochastic Particle Swarm Optimization approach. A novel iteration of particle swarm optimization (PSO) has been implemented to regulate the frequency and voltage of multi-area power systems^7^. The harmony search algorithm^8^ is a meta-heuristic optimization algorithm inspired by the musical improvisation process. It's used to solve optimization problems and has been adapted for tuning PID controllers in various control applications, including LFC. The study in Ref.^9^ applies the bat inspired algorithm (BIA) to optimize the gain values of the PI controller for LFC in a system consisting of two interconnected power networks. The hybrid firefly and pattern search technique are implemented to obtain the optimal gain values for PI and PID controllers in reference^10^. In Ref.^11^, a neuro-fuzzy hybrid intelligence based PI regulator was used for the LFC of an interconnected four-area power generating system. Several LFC-based optimization strategies have been introduced in the literature assessment of interconnected power systems, including classical, fuzzy logic, supervised artificial neural networks, artificial bee colony (ABC), and cuckoo search (CS). In Ref.^12^, a traditional PID controller with a population-based searching strategy known as the Falcon optimization algorithm is presented for the LFC study. The writers of the aforementioned studies examined the techniques for fine-tuning a PID controller and assessing its robustness via variations in load patterns and parameter uncertainty. The study^13^ introduces a new tuning approach called Virtual Time response-based iterative gain evaluation and re-design (V-Tiger) to iteratively tune PID gains for optimum control performance. The research^14^ examined the effectiveness of the quasi-oppositional harmony search algorithm based on predictive control in reducing frequency deviation. The performance of the suggested technique is evaluated by comparing it to the coordinated performance achieved using traditional controllers adjusted using the quasi-oppositional harmony search algorithm. References^15,16^ suggests a novel multi-objective tuning technique to enhance isolated micro-grid load frequency control (LFC) while accounting for the control signals from the micro-grid controller. In Ref.^17^, the load frequency issue of the two area interconnected power systems using solar and wind energy is covered.

To ensure the consistent frequency of a power system, it is necessary to incorporate an automated generation control (AGC) system with a reliable controller^18^. In Ref.^19^, the authors came up with the optimal controller for multi-area power system by using full-state feedback control approach. Reference^20^ presents a controller called the fractional order proportional tilt integral derivative plus One (FOPTD + 1) controller using global neighborhood algorithm to maintain a constant frequency. This controller has the capability to swiftly adjust the frequency during load disturbances.

### Literature on porpoising feature

The instability of porpoising in high-speed planning vessels was tackled by applying periodic force to mitigate the issue, without delving into the core causes of porpoising. Reference^21^ elucidated the mechanism and determinants responsible for the porpoising phenomenon during the takeoff of a flat plane. In Ref.^22^, the authors developed a dynamic model to depict the movement of seaplanes on the water’s surface. They also proposed a methodology for simulating and assessing seaplane porpoising. Reference^23^ examined the factors contributing to porpoising in high down-force race cars and proposed potential modifications to the car's setup to prevent it. The porpoising phenomena of a tunnel-type planning trimaran were investigated in Ref.^24^, using seven test scenarios. These scenarios were developed by considering the change in longitudinal locations of the center of gravity and the moment of inertia. The porpoising phenomena have only been explored and observed in vehicles operating in fluid medium like water so far. Table 1 gives a brief review on porpoising behavior.

**Table 1:** Brief review on porpoising behavior.

Authors/reference	Year of publication	Description/synopsis	Remarks
P. Gopi, and P. L. Reddy^2,25^	2015 and 2016	The coefficients of the LFC controller were acquired by the use of heuristic techniques such as Pattern search, Ant colony optimization, and Simulink design optimization The outcomes of the suggested LFC controller are contrasted with those of the standard PID controller	Simulation results were only addressed for a 10% change in the system's nominal parameters The porpoising aspect of the suggested PID controllers is not explored
M. R. Sathya, and M. M. T. Ansari^9^	2015	Developed a novel PID controller for LFC using nature inspired algorithm called Bat algorithm It has been shown that the suggested controller is less sensitive to variations in system parameters	The porpoising behavior of the proposed controller has not been discussed The impact of control actions on the system's response is not addressed
M. A. Ebrahim et al.^7^	2017	This work suggests the use of both I and PID controllers for LFC and AVR by using different variants of PSO The efficacy of the suggested PSO-based controllers for both small and large load disturbances in the presence of GRC is shown by a thorough comparison	
C. N. Sai Kalyan et al.^12^	2022	This research uses the Falcon Optimization Algorithm, a population-based search technique To improve system performance further, an AC line and a DC line are added in parallel to transfer bulk power during significant disruptions	
A. Kumar et al.^14^	2022	This research examined the effectiveness of the quasi-oppositional harmony search algorithm based on predictive control in reducing frequency error	
Q. J. Fu et al.^17^	2022	The load frequency issue of the two area interconnected power systems using solar and wind energy is covered	
M. H. N. Aliffrananda et al.^21^	2022	This research investigates the mechanism of porpoising and the factors that affect its occurrence	The reasons for the porpoising behavior were not specified
J. Liu, and F. Tian^22^	2023	The dynamics model of seaplane movements on the water's surface was created in this work, and a technique for simulating and evaluating seaplane porpoising was suggested	
L. Zan et al.^24^	2023	This research examines the porpoising phenomena seen in a tunnel-type planing trimaran. The porpoising effect is detected using the longitudinal stability limit curve	
A. E. Khalil et al.^15^	2023	It suggests a novel multi-objective tuning technique to enhance isolated micro-grid LFC	No information about porpoising feature
D. K. Sambariya et al.^26^	2023	Using the Fire Fly Algorithm, create the ideal load frequency controller for a single area system	There has been no debate regarding the porpoising behavior of the suggested controller There is no discussion of how control actions affect the system response (process variable)
Y. Güler and I. Kaya^27^	2023	Designed PI-PD controllers for a single-area, single- or multi-source power system's LFC The weighted geometric centre approach was used to determine the PI–PD controller parameters The effectiveness and robustness of the suggested PI–PD control system are assessed by taking into account the power system with nominal values as well as fluctuations in the system parameters	

## Research gap and contributions

Load frequency control (LFC) is a crucial aspect of power system operation, ensuring stability, reliability, and efficiency in the face of dynamic load changes and disturbances. Traditional LFC approaches often rely on heuristic tuning methods or simplistic control strategies, which may not fully exploit the system's dynamics or provide optimal performance under varying operating conditions. Moreover, the phenomenon of porpoising, characterized by oscillatory behavior in the system response, poses a significant challenge for LFC controllers, potentially leading to instability and degraded performance. Addressing these challenges requires advanced control techniques and systematic methodologies for controller tuning and porpoising detection.

This article makes several significant contributions to the field of power system control and optimization:

- Introduces a novel method for tuning Load Frequency Controllers (LFCs) using rat swarm optimization (RSO), leveraging the behavior of rats for efficient parameter exploration.
- Demonstrates through extensive simulations that the integration of RSO-based tuning leads to faster settling times, reduced overshoot, and enhanced transient stability in power grids, highlighting its effectiveness in optimizing system performance.
- Validates the robustness of the proposed approach across various load conditions and disturbances, contributing to the resilience and reliability of power systems under dynamic operating conditions.
- Addresses the critical issue of porpoising in LFC controllers, developing a framework to detect and mitigate this phenomenon, thereby improving system stability and performance.
- Advances the state-of-the-art in power system control by combining advanced optimization techniques with robust control strategies, offering valuable insights for the design and implementation of intelligent control systems in complex engineering applications.

The remainder of this article is as follows: Sect. "Load frequency control of thermal power generation station" discusses the model of the LFC of the thermal power generating station; Sect. "Tuning of LFC controller" covers a novel bio-inspired algorithm, called Rat Swarm Optimization, for optimal tuning of LFC controller gains; Sect. "Porpoising behavior of PID controller" discusses causes for figuring out the porpoising behavior of the LFC controller; in Sect. "Simulation results and discussion", the MATLAB simulation findings are described. Finally, this article concludes with research findings.

## Load frequency control of thermal power generation station

**Figure 1:**
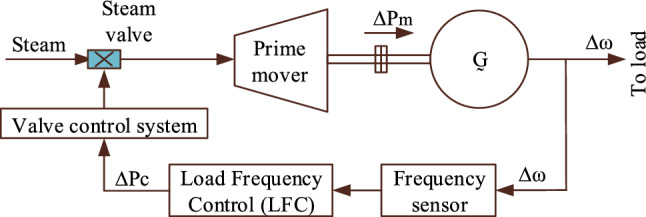
LFC of the TPGS—Schematic diagram.

Load frequency control (LFC) plays a pivotal role in the operation of power generating stations, serving to maintain a delicate equilibrium between power generation and demand in real-time scenarios. It constitutes a fundamental element of automatic generation control (AGC) systems, which are tasked with the continuous adjustment of generator power outputs to uphold system frequency within predefined acceptable bounds^28^. This dynamic regulation is indispensable for the overall stability and reliability of power grids, particularly in the face of fluctuating loads and unforeseen disturbances. Central to LFC is the coordination of control actions to counteract deviations from nominal operating conditions, ensuring that frequency variations remain within prescribed limits to safeguard the integrity of the electrical network. Figure 1 illustrates the schematic diagram of the Load Frequency Control system implemented in a single-area thermal power generating station (TPGS), providing a visual representation of the control mechanisms involved in maintaining system stability and frequency regulation.

The dynamics of a power generating station, in terms of frequency deviations, can be analyzed by developing its transfer function model^25^. In this work, the transfer function model of the thermal power system is derived based on the governing equations of the system, which include the dynamic response of the speed governor, steam turbine, and power generating station with load.

### Speed governor model

The speed governor in the LFC plays an important role in regulating the turbine-generator output power to maintain system frequency within acceptable limits. The dynamics of the speed governor, using a transfer function model, describe how the governor responds to the changes in system frequency and adjusts the turbine output power accordingly^26^. The speed governor mechanism acts as a comparator whose output *∆P*_*v*_*(s)* is the difference between the reference set point *∆P*_*ref*_*(s)* and power *∆ω(s)/R*. Assuming the linear relationship and time constant (T_sg_), the transfer function model of the speed governor is represented as,

1 $$ {\text{Gsg(s)}} = \frac{{\Delta {\text{Pv(s)}}}}{{\Delta {\text{Psg(s)}}}} = \frac{{1}}{{\text{1 + sTsg}}}{.} $$

### Steam turbine model

The turbine is used to produce the mechanical power for a given steam input. The turbine is also known as the prime mover. The dynamic model of the steam turbine relates the change in mechanical power generated *∆P*_*m*_*(s)* to the change in steam valve position *∆P*_*v*_*(s)*. The turbine time constant (T_st_) is used to approximate the non-reheat type steam turbine transfer function model^26^.

2 $$ {\text{Gst(s)}} = \frac{{\Delta {\text{Pm(s)}}}}{{\Delta {\text{Pv(s)}}}} = \frac{{1}}{{\text{1 + sTst}}}. $$

### Model of power generation station with load

The thermal power generating station mainly consists of an AC generator. The swing equation is used to get the AC generator's transfer function model. Power station loads may have various electrical properties. For instance, there are lighting and heating loads that have a power consumption that does not depend on frequency, as well as rotating loads whose power consumption changes with frequency. The sensitivity of the rotating loads depends on the speed-load features of the rotating devices. Let *H* be the rotational inertia of the AC generator and rotating loads, *∆δ* is the change in rotor angle, ω represents the nominal frequency, and *∆P*_*e*_ is the electrical power consumed by the loads, and *∆P*_*m*_ is the mechanical power developed by the turbine. The mathematical representation of the swing equation is

$$ \frac{{2{\text{H}}}}{\omega }\frac{{d^{2} }}{{dt^{2} }}\Delta \delta = \Delta {\text{Pm}} - \Delta {\text{Pe}}. $$

Simplifying and taking Laplace transform,

3 $$ {\text{s}}\Delta \omega \left( {\text{s}} \right) \, = \frac{1}{{2{\text{H}}\omega }}\left( {\Delta {\text{Pm}}({\text{s}}) - \Delta {\text{Pe}}({\text{s}})} \right), $$

The change in electrical power consumed by the loads *∆P*_*e*_ = *∆P*_*Ld*_*(s)* + *D∆ω(s).* Where *∆P*_*Ld*_ is the change in load that is independent of frequency variations, *D∆ω(s)* is frequency-dependent load change, and D is represented as the ratio of percentage change in load to percentage change in frequency^26^. Substituting *∆P*_*e*_ in (3) and rewriting,

4 $$ \frac{{\Delta \omega {\text{(s)}}}}{{\Delta {\text{P}}_{{\text{m}}} {\text{(s)}} - \Delta {\text{P}}_{{{\text{Ld}}}} {\text{ (s)}}}} = \frac{1}{{2{\text{Hs}}\omega + {\text{D}}}} = \frac{{{\text{Kgs}}}}{{\text{sTgs + 1}}}, $$

where K_sg_ is the generating station gain constant and T_sg_ represents the time constant.

**Figure 2:**
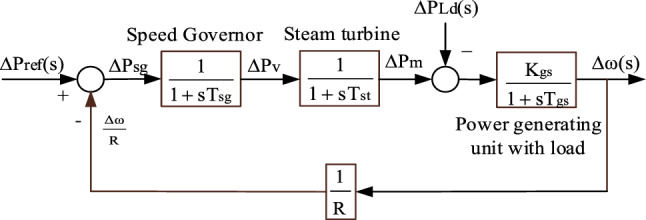
Transfer function model of single area TPGS.

The transfer function model of the single area TPGS is obtained by combining Eqs. (1), (2), and (4) as seen in Fig. 2.

The transfer function relating the deviation in load demand to the change in frequency is given by,

5 $$ \frac{{\Delta \omega ({\text{s}})}}{{ - \Delta {\text{P}}_{{{\text{Ld}}}} ({\text{s}})}} = \frac{{\left( {{\text{TsgTsts}}^{{2}} {\text{ + (Tsg + Tst) + 1}}} \right){\text{Kgs}}}}{{\left( {{\text{TsgTsts}}^{{2}} {\text{ + (Tsg + Tst) + 1}}} \right)\left( {\text{sTgs + 1}} \right){\text{ + Ksg/R}}}}. $$

Here R is the turbine speed droop. The steady state deviation in frequency, for the step load change (∆P_Ld_), is obtained by applying the final value theorem to the above equation i.e.

6 $$ \omega_{ss} = \mathop {\lim }\limits_{s \to 0} {\text{s}}\Delta \omega ({\text{s}}) = - \Delta P_{Ld} \frac{{{\text{Kgs}}}}{{1 + {\text{Ksg}}/{\text{R}}}}, $$

**Figure 3:**
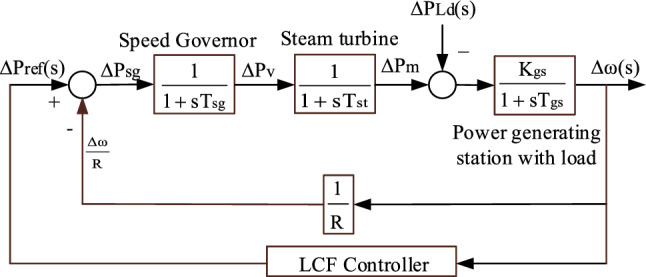
Transfer function model of single area TPGS with LFC controller.

The oscillations and steady-state deviation in the load frequency can be reduced by utilizing a controller known as the LFC controller. The LFC controller will continuously modify the power generation in response to frequency deviations and minimize the frequency error^29^. Figure 3 shows the single area TPGS with an LFC controller.

## Tuning of LFC controller

A proportional-integral-derivative (PID) controller is a widely used control mechanism that stabilizes the system and minimizes the oscillations by modifying the control signal according to the discrepancy between the desired set-point and the actual output. A PID controller is used as an LFC controller in this study. To achieve optimal control performance, stability, and robustness in various applications, the PID controller must be tuned^30,31^. In this work, rat swarm optimization (RSO) is proposed to obtain the optimal PID parameters, and the results were compared with Zeigler-Nichols and Firefly Algorithm-based PID controllers.

### Rat swarm optimization

The rat swarm optimization (RSO) is an innovative bio-inspired meta-heuristic algorithm designed for solving global optimization challenges. Rats often exhibit very aggressive behavior, which may lead to the death of other animals. The main motivation for this method is this aggressive behavior while hunting and wrestling with prey^32^.

7 $$ x_{i} = \, b_{il} + \, rand \times \left( { \, b_{iu} - \, b_{il} } \right), $$

where i = agent and it is represented as 1, 2, 3, . . . . N, *b*_il_ and *b*_iu_ are the lower and upper bounds for ith agent, and N is the total number of agents.

Based on the social agonistic behavior of rats, the movement of each rat in the swarm as influenced by the presence of the prey can be defined. To establish this behavior mathematically, the assumption that the optimal search agent is aware of the prey’s position is used. Considering the optimal search agent found so far, the remaining search agents may update their positions accordingly. The updated position of the ith rat (*P*_*ui*_(*x*)) can be evaluated using the following mathematical equation:

8 $$ {\text{P}}_{{{\text{ui}}}} \left( {\text{x}} \right) \, = \, \alpha .{\text{P}}_{{\text{i}}} \left( {\text{x}} \right) \, + \, \lambda .\left( {{\text{P}}_{{\text{b}}} \left( {\text{x}} \right) \, - {\text{ P}}_{{\text{i}}} \left( {\text{x}} \right)} \right), $$

where *P*_*i*_*(x)* is the ith rat position, *P*_*b*_*(x)* is the optimal position obtained so far, the parameters *α* and *λ* are the random values.

The values of *α* and *λ* can be found using,

9 $$ \alpha \, = \, \gamma \, - x \frac{\gamma }{Itrmax}\,\,{\text{and}}\, \lambda \, = { 2}.{\text{rand}}() $$

where γ is the random value, *x* is the current iteration and is given by 1, 2, 3, . . . *Itr*_*max*_, the random values of *λ* and *γ* are in search space [0, 2] and [1, 5] respectively.

The wrestling with prey behavior of the rats can be mathematically described to generate the updated new position of the rat, *P*_*i*_*(x* + *1).*

10 $$ \left| {Pb(x) - [\alpha .Pi(x) + \lambda .(Pb(x) - Pi(x))]} \right|. $$

Equation (10) modifies the positions of the search agents and stores the optimal solution. The values of *α* and *λ* are responsible for exploration and exploitation during the entire iteration process.

#### Implementation of RSO

The following steps are employed to adjust the PID settings based on the system's response using the RSO algorithm.

1. Initialize the population of rats N (each rat represents a set of PID parameters), maximum iterations, termination criteria, rat’s position, parameters *α*, *γ*, and *λ*.
2. Evaluate the fitness of each search agent (i.e. rat) in the swarm by minimizing the following objective function (J). This approach uses an objective function based on the integral time absolute error.
  11 $$ {\text{J}}_{{{\text{ITAE}}}} = \int\limits_{0}^{{{\text{ts}}}} {{\text{t}}\left| {\Delta \omega } \right|dt} $$
3. Revise the location of each rat by considering its current position (x_i_), using (7). Determine the best possible solution (*P*_*b*_*(x)*) by the swarm.
4. During each iteration, while *x* < *Itr*_*max*_, update the position of each rat (i.e. search agent) using (8), and update the parameters *α*, *γ*, and *λ* using (9).
5. Assess the fitness of every rat. Modify any search agent that exceeds its specified boundaries. Again, estimate the fitness of each updated rat. Then update the current optimal solution with the previous optimal solution, if any.
6. Verify the termination condition, i.e. *x* < Itr_max_. If the condition is met, go to the next step. Otherwise, iterate the process of evaluating fitness, updating position, and local and global searches until a termination criterion is met.
7. Consider the current solution as the optimal solution. Display the optimal solution (as optimally tuned PID values) and its fitness value.

**Figure 4:**
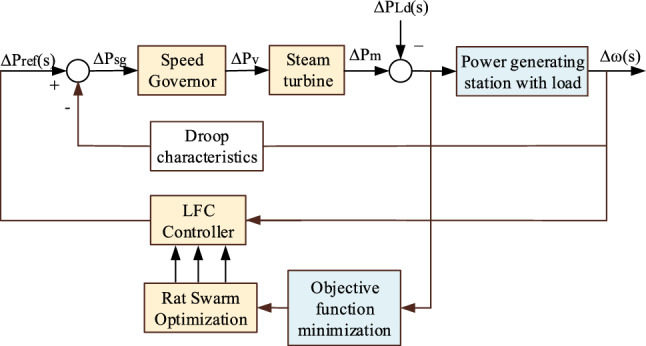
Implementation of suggested algorithm on LFC.

The procedure for applying the suggested algorithm to LFC to get the LFC controller coefficients is shown in Fig. 4. The objective function (11) is minimized for the optimal LFC controller tuning, taking into account the following limitations: *K*_*pl*_ < *K*_*p*_ < *K*_*pu*_; *K*_*il*_ < *K*_*i*_ < *K*_*iu*_; and *K*_*dl*_ < *K*_*d*_ < *K*_*du*_. Also, the derivative filter time constant (*t*_*f*_) < 0.01. To determine the optimal coefficients, 25 simulation runs were performed due to its stochastic nature. Every run has a population size of 50 and a maximum of 100 iterations. The suitable value of γ, in the search space [1, 5], is randomly chosen.

**Figure 5:**
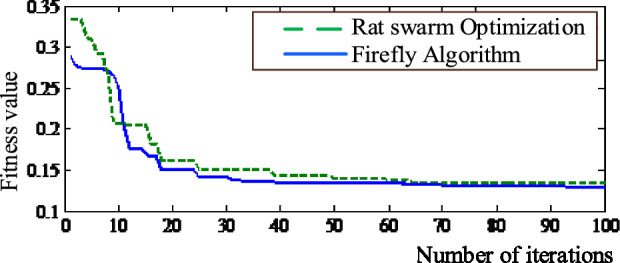
Convergence comparison of firefly and rat swarm optimization.

Figure 5 compares the convergence of the firefly method with rat swarm optimization. It is clear from the graphic that the rat swarm optimization outperforms the Firefly algorithm in terms of fitness values.

### Algorithms for comparison

In this study, the outcomes achieved by rat swarm optimization (RSO) are compared with the Zeigler-Nichols and Firefly algorithms.

#### Firefly algorithm

The firefly algorithm is a meta-heuristic optimization algorithm inspired by nature that solves optimization problems by imitating the flashing activity of fireflies. It was introduced in 2008 by Xin-She Yang. This algorithm has become popular because of its adaptability and efficiency in resolving a variety of optimization issues. This approach is used to achieve optimum tuning of a PID controller in the following manner.

**Figure 6:**
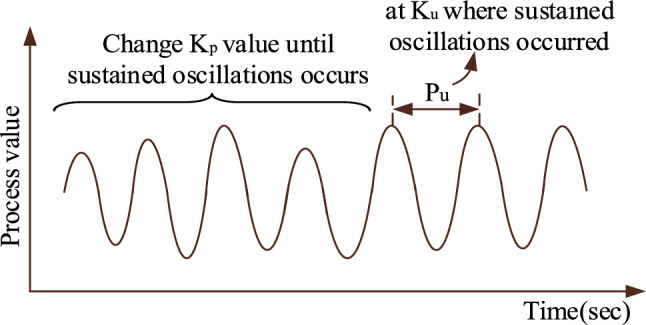
Closed loop response of plant with P control action only.

Randomly distribute a population of fireflies around the search space. Assign a brightness, which represents the objective function value, to each firefly depending on its location. Every firefly in the population is drawn towards brighter fireflies and pushed away from dimmer ones. The attractiveness of a firefly is determined by its brightness and the distance from the other firefly. Fireflies move towards brighter fireflies by altering their locations in a process similar to a random walk. Once it has moved, adjust each firefly's brightness according to its new location. Based on their brightness levels, choose the optimal firefly (solutions). Repeat this process until the termination condition is met. Terminate the algorithm when the termination criterion is satisfied^26^.

#### Ziegler-Nichols (ZN) closed loop method

This method is a widely used empirical methodology for adjusting PID controllers. It offers a simple method for determining the proportional, integral, and derivative gains and is based on the system's response to step changes^33^. The following steps are used to optimize the PID coefficients^13^.

**Table 2:** PID gains obtained from various algorithms.

Controller type	Proportional gain (K_p_)	Integral gain (K_i_)	Derivative gain (K_d_)
Rat swarm optimization (proposed algorithm)	1.456	2.158	0.451
Firefly algorithms^26^	5.7586	4.5378	1.5689
Ziegler-Nichols closed loop method	3.5916	7.172	0.45

1. Set the integral and derivative gains to zero. Increase the proportional gain until the system starts to oscillate steadily, as shown in Fig. 6. From the sustained oscillations, measure the ultimate gain (K_u_) and the corresponding oscillation period is T_u_.
2. Based on the ultimate gain and oscillation period, the PID controller gains are evaluated as *K*_*p*_ = *0.6K*_*u*_; *K*_*i*_ = *1.2K*_*u*_*/ T*_*u*_, and *K*_*d*_ = *0.075K*_*u*_*T*_*u*_. After successfully applying these tuning approaches to the single area TPGS, the optimum PID gains are shown in Table 2.

## Porpoising behavior of PID controller

Porpoising refers to describing the oscillatory behavior of a process variable (PV) as it approaches the set point (SP) after a sudden change in the set point. Porpoising is usually considered undesirable behavior for a feedback loop since it combines the adverse effects of over-tuning (leading to instability) with the undesirable effect of under-tuning (delay in reaching the desired set-point). This feature can only be caused by two control actions: proportional and derivative. Integral action cannot have the capability to cause porpoising. The following are the potential problems that may occur in real-world situations as a result of porpoising:

- Porpoising can destabilize the system by causing excessive oscillations. These oscillations have the potential to magnify over time, resulting in instability if not addressed.
- It has the potential to negatively impact the system's accuracy, response time, and overall performance. It can have a major effect on performance metrics in systems where precise control is necessary.
- The mechanical components of the plant can experience more stress due to oscillations generated by porpoising, which causes mechanical problems in industrial systems.
- Porpoising may cause instability in the controller. This impact may be exacerbated if the controller's gain is not adequately set to mitigate oscillations.

### Identifying porpoising feature

The main reasons why porpoising conditions arise in PID controllers are the location of complex poles in the s-plane, the low damping ratio of closed-loop poles, and P and D control actions of the PID controller.

#### Based on location of poles on s-plane

According to control engineering, a plant/system is considered stable if every pole is located on the left side of the s-plane. The location of complex poles in the s-plane also affects the stability of the system. Specifically, the negative real part of the complex poles indicates plant stability and the imaginary part represents oscillations in the plant response. More imaginary part magnitude results in more oscillations in the plant response, which might cause the controller to porpoise. The system exhibits improved transient response when the poles are diverging from the imaginary axis.

#### Based on damping factor

**Figure 7:**
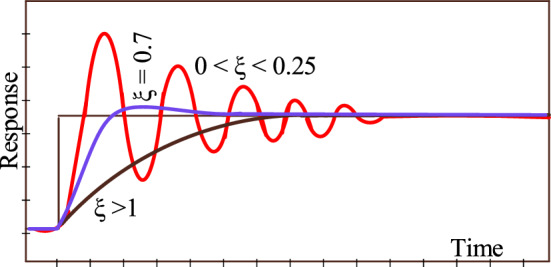
Damping ratio vs Plant response.

In a control system, the oscillations in the plant’s response are influenced by the damping ratio, i.e. it quantifies how oscillations decay over time in a system's response to a step input. The oscillation in plant response for the given value of the damping ratio is depicted in Fig. 7.

According to Fig. 7, the response reaches the steady state without oscillation in the shortest possible time, minimizing both rise time and settling time when the damping ratio reaches unity. The controller becomes less porpoising as a consequence. At lower damping ratios (generally 0 < *ξ* < 0.25), the plant exhibits more noticeable oscillations with slower decay. As a consequence, the controller is more vulnerable to porpoising.

#### Based on P and D control actions of controller

**Figure 8:**
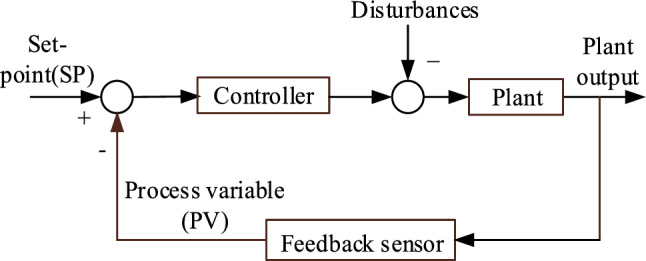
Closed loop plant with controller.

Consider a closed-loop plant in Fig. 8. The present measured value of a part of a process that is being monitored or controlled is referred to as the process variable (PV). The desired value for the PV is termed as a set point (SP).

**Figure 9:**
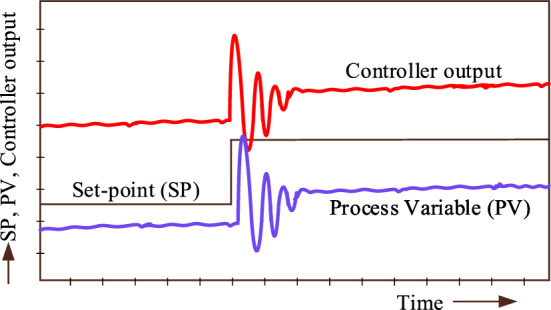
P control action only.

The porpoising or oscillations in the PV might result from either P or D control actions, or, if the controller is very aggressive, it could result from all control operations. Comparing the trend of the PV and the controller output is one method of determining which control action might cause porpoising. By contrasting the two trends, examine the phase difference between the PV and loop controller output to determine which action is more prominent. For example, take a look at Fig. 9, where the type controller displaying the trends is a reverse-acting controller since the controller output rises in response to a rise in the set-point (SP) (implying that the controller output falls in response to a rise in PV). This conduct is contrary to that of a direct-acting controller.

The controller tuning, in Fig. 9, is unsuitable since there exists a significant deviation between the PV and the set point. This indicates that the controller is set up for aggressive proportional action. The phase shift between the PV and controller output during the oscillations is observed as + 90° i.e. the controller output peaks lead the PV peaks. This indicates that the controller is set up for aggressive derivative action.

**Figure 10:**
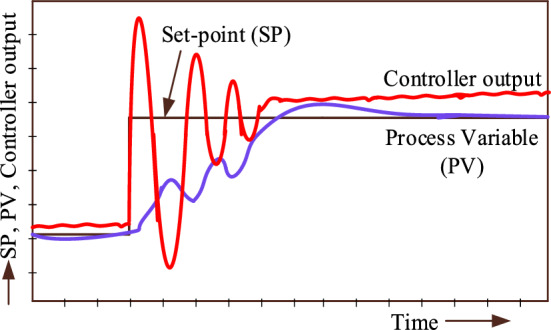
PID control actions.

In Fig. 10, the controller output step-up in response to a rise in the set-point SP or the controller output step up in response to a rise in PV. The comparison of trends indicates that the controller tuning is too aggressive and there is porpoising in PV. This porpoising is due to P and D control actions. Observe the phase shift between the PV and controller output during the oscillations, the controller output peaks lead PV peaks. This indicates that the D control action is too aggressive.

## Simulation results and discussion

Consider a 3-Phase, 50 Hz, 600 MW non-reheat type thermal power generating station with a 1% sudden change in load demand (i.e. 6 MW change in load demand). The parameters used in Fig. 2 and their nominal values are listed in Ref.^26^. MATLAB 2022a was used to build the proposed power station, and the simulation results for a 1% change in load demand were examined.

### Dynamic response analysis

**Figure 11:**
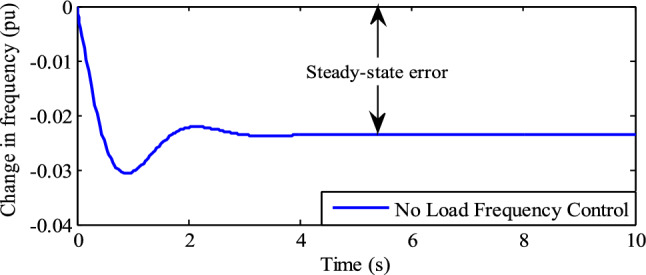
Frequency variations without LFC.

Figure 11 shows the frequency variation of the single area TPGS without an LFC controller. It indicates that in the absence of an LFC controller, the change in frequency of the power plant exhibits a negative peak-overshoot of 0.0307 pu (or 1.535 Hz) and a steady-state frequency error of 0.0235 pu (or 1.175 Hz) with a settling time of 4.65 s. In practice, the frequency error will cause damage and malfunction of electrical loads (such as rotating devices, transformers, and frequency-dependent loads), and lead to power station instability (potentially causing power blackouts). To restrict these limitations, a LFC controller is needed. In this work, a PID controller tuned by different algorithms is used as a LFC controller.

**Figure 12:**
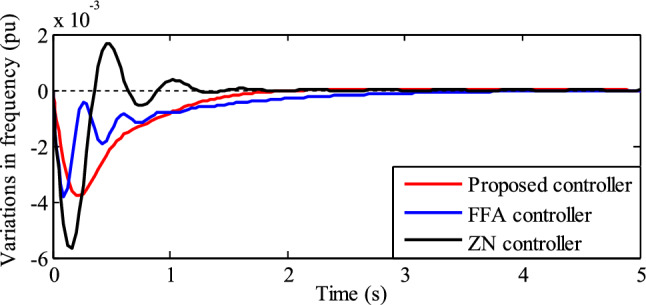
Frequency variations with LFC.

A comparison of frequency fluctuations including LFC is shown in Fig. 12. It indicates that the settling time and negative peak overshoot with the ZN controller acting as supplemental control are 1.51 s and 0.05665 pu (or 2.842 Hz), respectively. Comparably, the FFA controller has a zero steady-state error and a negative peak overshoot of 0.038 pu (or 1.90 Hz) with a settling time of 4.50 s. In addition, while utilizing the RSO-based PID controller, there is no steady-state error, a negative peak overshoot, and a settling time of 0.0378 pu (or 1.89 Hz) and 1.98 s, respectively.

The performance of the frequency variations in TPGS with various LFC (PID) controllers, as shown in Figs. 11 and 12, is summarized in Table 3.

**Table 3:** Performance comparison of various LFC controllers.

Type of LFC controller	Negative peak overshoot		Frequency variations		Settling time
	pu	Hz	Pu	Hz	Sec
No controller	0.0307	1.535	0.0235	1.175	4.65
Proposed controller	0.0379	1.895	0	0	1.98
FFA controller^26^	0.0380	1.90	0	0	4.50
ZN controller	0.05665	2.842	0	0	1.51

Table 3 shows that, when compared to other controllers, the RSO PID controller performs better in transient conditions in terms of zero frequency error, low negative peak overshot, and settling time. The closed-loop poles and their corresponding damping factors for different PID controllers are listed in Table 4.

**Table 4:** Closed loop poles and their damping factors for different LFC controllers.

Parameters	Type of LFC controller					
	Rat swarm optimization (proposed algorithm)		Firefly algorithm^26^		ZN method	
Closed loop poles	− 1.98 ± 0.04*i*	− 5.98 ± 8.18*i*	− 1.01, − 2.91	− 5.98 ± 18.7*i*	− 3.77 ± 2.96*i*	− 4.17 ± 9.82*i*
Damping factor	0.967	0.588	1	0.305	0.787	0.391

**Figure 13:**
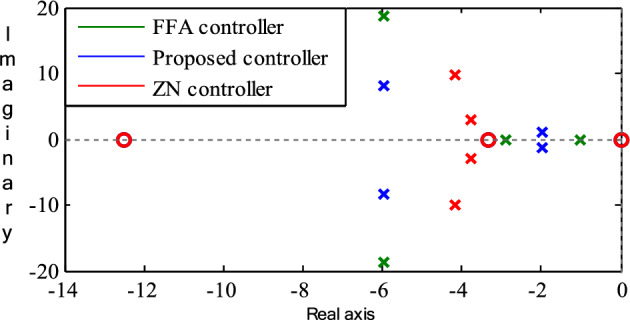
Location of poles on s-plane.

The location of the poles of single-area TPGS with different LCF controllers is illustrated in Fig. 13. With the proposed controller, there are fewer oscillations in the frequency response of single-area TPGS because all of the poles are located far from the origin. Despite having two real poles, the single area TPGS with FFA controller frequency response is more oscillatory because of its − 5.98 ± 18.*7i* poles and its lower damping factor. Similar to this, the complex nature of the poles and the lower damping factor of the single-area TPGS with ZN controller cause an oscillating frequency response. This discussion concludes that the single area TPGS with the proposed controller is more stable than the other controller.

**Figure 14:**
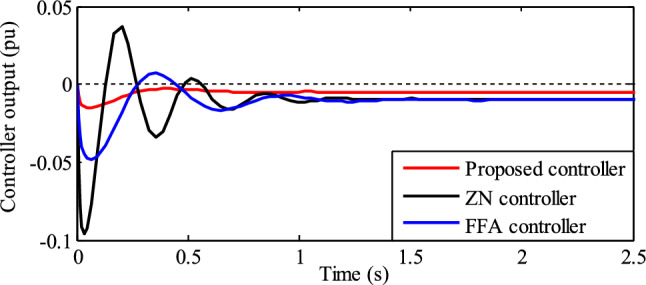
Comparison of a control signal using various PID controllers.

The controller output refers to the signal generated by the LFC based on the comparison between the actual frequency of the system and its reference frequency. A comparison of the control signal using various PID controllers is shown in Fig. 14. When compared to other controllers, the RSO PID controller exhibits negligible oscillations in the control signal.

**Figure 15:**
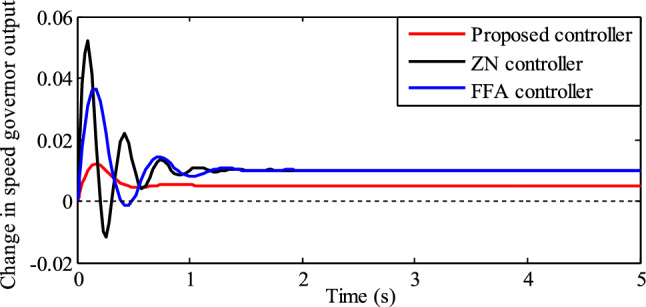
Comparison of speed governor output.

The change in speed governor output, using different PID controllers, is depicted in Fig. 15. The variations in the speed governor output have a direct effect on turbine output power.

These variations in turbine power can affect the overall balance of supply and demand at the power generating station. Based on Fig. 15, the fluctuations in the speed governor output with the proposed PID controller are negligible. As a result, the power generating station with the proposed controller demonstrates greater stability.

### Recognizing porpoising PID controller

As mentioned in section "Porpoising behavior of PID controller", the porpoising characteristic of the PID controllers used in this article is recognized by the location of poles in the s-plan and their damping ratio; P and D control actions of the PID controller.

#### Based on the location of poles on s-plane and its damping factor

If the damping ratio of the complex poles generated by the plant/system in conjunction with the PID controller is low, then the PID controller is more prone to porpoising. Let us examine the damping ratio values shown in Table 4. When using the Firefly controller, the real poles − 1.01 and − 2.91 have a unity damping ratio, while the complex pole − 5.98 ± 1.87*i* has a damping ratio of 0.305. Similarly, the complex pole − 5.98 ± 1.87*i*, produced by the proposed controller, has a damping ratio of 0.588, and the other complex pole − 1.98 ± 0.04*i* has a 0.967 damping ratio. According to this discussion, the Firefly controller is more prone to porpoising than the RSO PID controller since the Firefly controller has a lower damping ratio. The porpoising effect in the controller can be minimized by improving the damping factor.

#### Based on P and D control actions of controller

Assume that the TPGS frequency changes are the process variable (PV). The PID controller's porpoising feature is the oscillations in the PV at a particular set-point, and the controller's goal is to achieve a steady state frequency error of zero. Monitor the controlled output response (PV) and look for repetitive patterns of overshooting and undershooting before it reaches its steady state.

**Figure 16:**
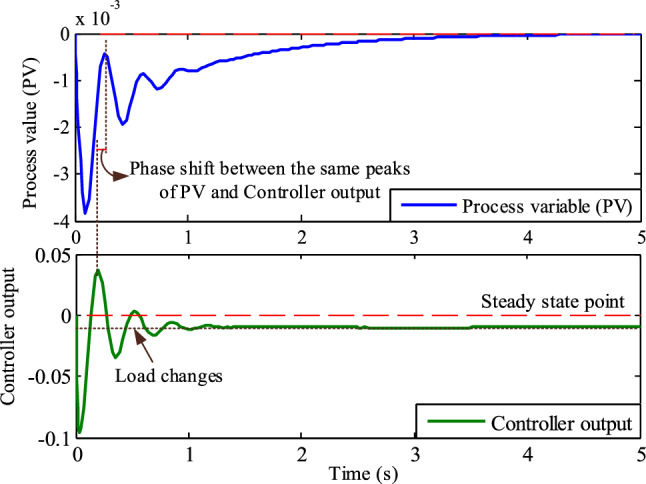
Comparison of identical peaks of the controller output and process variable for phase shift (Firefly algorithm-based PID controller).

The fluctuations in PV and controller output with the Firefly-based PID controller are shown in Fig. 16. This figure shows that before the PV reaches its steady state, there are more oscillations in the process variable due to the aggressive proportional action of the controller. It is evident from looking at the identical peaks of the controller output and process variable in Fig. 16 that the controller output wave is 90^0^ ahead of the PV wave. This is due to an aggressive derivative action. According to this discussion, the Firefly algorithm-based PID controller is referred to as a porpoising PID controller.

**Figure 17:**
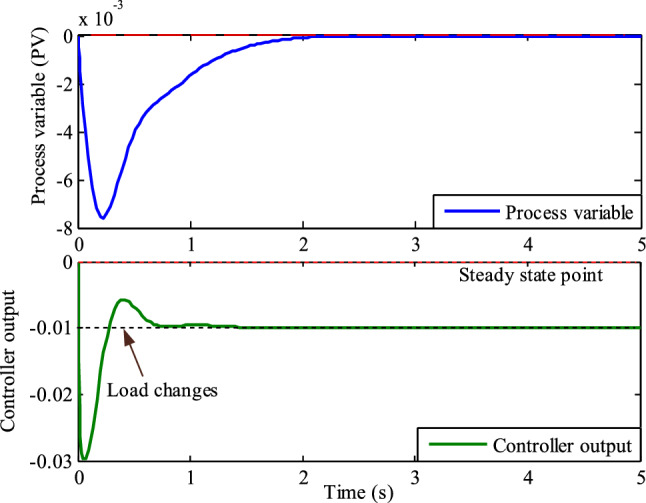
Comparison of identical peaks of the controller output and process variable for phase shift (Rat swarm optimization-based PID controller).

Figure 17 illustrates the process variable and controller output of TPGS with RSO based PID controller. This figure demonstrates that the controller tuning is satisfactory since there are no oscillations in the process variable before it reaches a steady state. Upon analyzing the phase changes between the controller output and process variable, it is evident that there is a presence of derivative action. This is shown by the fact that the controller output leads the PV during oscillation. This implies that the PID controller with integral action and a suitable amount of proportional action provides a quick response to changes in load.

## Conclusions and future research directions

This article presents a new and effective way for optimizing the tuning of a PID controller used in load frequency control of a single-area TPGS. The causes of the porpoising effect in the PID controller were also discussed. The dynamic load frequency analysis of the TPGS is carried out for a load disturbance of 1%. The frequency variations of the TGPS with RSO-based PID controller are compared to those of the Firefly algorithm and Zeigler Nichols-based PID controllers. The TGPS with RSO-based PID controller has been shown to be better and more stable compared to the PID controller.

The porpoising phenomena in the PID controller was found to be caused by the position of the poles on the s-plane and their damping ratio, as well as the P and D control actions of the PID controller. The porpoising phenomenon was examined by analyzing these components using MATLAB simulation. According to the simulation investigation, the PID controller tuned via Rat Swarm Optimization is more resistant to porpoising compared to other controllers.

The future work of this research is implementing the model in a real-time environment to include subjective factors. Also, identify the porpoising PID controllers that are involved in various real-time applications such as tank level control, EV drives, Automatic Voltage Regulators (AVRs), AGC of multi-area and multi-source power system. Future research directions in this field could also explore several avenues to further enhance the effectiveness and applicability of Load Frequency Control (LFC) systems. One promising direction is the investigation of advanced optimization algorithms beyond Rat Swarm Optimization (RSO), such as evolutionary algorithms or machine learning approaches, to fine-tune LFC controllers and adapt them to diverse operating conditions. Additionally, exploring the integration of renewable energy sources into power systems presents an important area for future study, as it requires novel control strategies to maintain grid stability in the presence of intermittent generation. Furthermore, the development of decentralized or distributed control schemes could offer scalability and resilience to LFC systems, particularly in large-scale power networks. Finally, conducting hardware-in-the-loop simulations to validate and execute the suggested control techniques in real-world power systems would provide vital insights into their practical performance. In summary, future research should focus on tackling the changing obstacles and needs of contemporary power systems, eventually contributing to the progress of sustainable and robust energy infrastructure.

## Data Availability

The datasets used and/or analyzed during the current study are available from the corresponding author upon reasonable request.
